# Within-host evolution of *Brucella canis* during a canine brucellosis outbreak in a kennel

**DOI:** 10.1186/1746-6148-9-76

**Published:** 2013-04-12

**Authors:** Miklós Gyuranecz, Brandy D Rannals, Christina A Allen, Szilárd Jánosi, Paul S Keim, Jeffrey T Foster

**Affiliations:** 1 Institute for Veterinary Medical Research, Centre for Agricultural Research, Hungarian Academy of Sciences, Budapest, Hungária körút 21, 1143, Hungary; 2 Center for Microbial Genetics & Genomics, Northern Arizona University, 1298 S. Knoles Drive, Flagstaff, AZ, 86011-4073, USA; 3 Veterinary Diagnostic Directorate, National Food Chain Safety Office, Budapest, Tábornok utca 2, 1143, Hungary

**Keywords:** *Brucella canis*, Brucellosis, Evolution, MLVA, VNTR

## Abstract

**Background:**

Little is currently known about *Brucella* evolution within the host during infection. The current study is the first to employ fine-scale genotyping on an isolate collection derived from a *Brucella canis* outbreak. Eight isolates of *B. canis*, cultured from different tissues of three dogs (female, stud dog, puppy of another female) from a single kennel over three months were genetically characterized with a 15-marker multi-locus, variable-number tandem repeat (VNTR) analysis (MLVA) to assess the genetic relatedness of isolates and potential rapid mutational changes.

**Results:**

MLVA discriminated among the otherwise indistinguishable isolates from different animals and from isolates collected at different time points within each host, with different VNTR alleles being detected at multiple dates and tissue sites. We suspect that all isolates cultured from the female, puppy, and stud dogs originated from the same strain, with subsequent rapid *in vivo* mutations. However, high mutation rates and apparent in several of the loci prevented making definitive epidemiological relationships among isolates.

**Conclusions:**

This investigation highlights the rapid *in vivo* genetic mutations of several VNTRs of *B. canis* over a short time period in the host and the emergence of alternate alleles. However, this work also suggests the challenges of using highly mutable VNTRs to infer epidemiological relationships of strains within a short duration outbreak.

## Background

*Brucella canis*, the etiologic agent of canine brucellosis, can lead to severe economic loss in infected kennels. Canine brucellosis is a contagious disease transmitted via contaminated reproductive, oronasal, or conjunctival fluids [1]. The bacteria are particularly associated with reproductive tissue, causing abortion in females and epididymitis and prostatitis in males [2]. *Brucella canis* affects human beings, but few cases are reported in humans [1,3] or other animals, suggesting either host-preference for dogs or limited opportunities for transmission to other species.

Several multi-locus, variable-number tandem repeat (VNTR) analysis (MLVA) systems have been developed and described for *Brucella* species [4-7]. MLVA is a rapid, reproducible molecular typing system providing high discriminatory power among closely related strains [8,9]. These characteristics make MLVA a potentially powerful tool for epidemiological (e.g. to follow local epidemics) and forensic investigations of events involving *Brucella* species [10-14] and many other bacterial agents. This molecular approach specifically allows for fine-scale detail on likely source populations of new brucellosis cases in human cases [14-16] and tracking outbreaks in wildlife and livestock [7,17].

The first known outbreak of *B. canis* infection in Hungary was described in 2009 [18]. The affected kennel population consisted of 31 dogs of various ages. The disease was most likely introduced to the kennel by a pregnant female whose abortion was the first obvious reproductive disorder in October 2008 [18]. Sporadic reproductive clinical symptoms were detected by the breeder during the eight months preceding the first laboratory examination.

The aim of this study was to genetically characterize the eight strains of *B. canis* isolated from three dogs in a kennel during three months of the outbreak. Our goal was to use MLVA to assess the level of within-host genetic variation at different dates and sampling sites on the dogs for a better understanding of rapid *Brucella* evolution and its use for epidemiology.

## Methods

Samples of blood, vagina and throat swabs and lymph nodes (mesenteric and sublingual lymph nodes) were obtained from every resident dog at 1-month intervals in June, July and August 2009. All experiments were performed in accordance with all applicable institutional and national guidelines and regulations and with the consent of the dog owner. The samples were collected from tissues typical for clinical brucellosis investigations and submitted for bacteriological examination as described previously [18]. Eight *B. canis* isolates (Bc 1-8) were recovered from three animals on selective *Brucella* agar plates (Oxoid Ltd., Cambridge, United Kingdom) (Table 1). Primary isolates were subcultered on trypticase soy agar plates (Difco, BD Diagnostic Systems, Sparks, MD) three times to reveal pure culture before DNA extraction (QIAamp DNA Mini Kit, Qiagen Inc., Valencia, CA).

**Table 1:** Summary of host, isolation date, source and primary and alternate VNTR alleles of the studied strains

				**Polymorphic VNTR**							**Monomorphic VNTR**										
**Isolate ID**	**Host**	**Isolation date**	**Isolation source**	**1**	**1 alt**	**2**	**2 alt**	**30**	**33**	**33 alt**	**3**	**7**	**14**	**16**	**20**	**21**	**25**	**27**	**28**	**29**	**31**
Bc 1	female, abortion in June	June	foetus	234		214		568	295	303	417	306	121	233	439	100	490	413	198	260	507
Bc 2		July	blood	234	242	206	214	568	303		-	306	121	233	439	100	490	413	198	260	507
Bc 3		July	vagina	234		206		568	263		417	306	121	233	439	100	490	413	198	260	507
Bc 4	stud dog, without clinical symptoms	July	blood	234		214		568	303	311	417	306	122	233	439	100	490	413	198	260	507
Bc 5		August	lymph node	234		231		568	295		417	306	121	233	439	100	490	413	198	260	507
Bc 6	female puppy, born with infection in March	July	blood	242	250	150	214	574	255	263	417	306	121	233	439	100	490	413	198	260	507
Bc 7		August	blood	234		214		568	255	263	417	306	121	233	439	100	490	413	198	260	507
Bc 8		August	lymph node	234	242	214		568	255	263	417	306	121	233	439	100	490	413	198	260	507

Species identity for all isolates was confirmed by real-time PCR by a *B. canis*-specific assay [19]. The eight isolates were screened with a 15-marker MLVA system as previously described [5], with modifications. We redesigned the three largest VNTR primer sets (VNTRs 2, 29, 33) to create smaller amplicons to reduce this potential source of allelic variation (Table 2). Larger amplicons generally exhibit greater variation in sizing due to loss of linearity for ladder fragment standards above ~500 bp. We ran these new primers in 10 μl reactions in singleplex under the following conditions: 1× PCR buffer, 2 mM MgCl_2_, 0.2 mM dNTPs, 0.04 U platinum Taq DNA polymerase (Invitrogen, Carlsbad, CA), 0.10 μM forward primer and 0.08 μM reverse primer. Thermocycling parameters were an initial denaturation at 94°C for 5 min, followed by 94°C for 45 s, 65°C for 45 s and 72°C for 45 s for 35 cycles, and a final annealing step at 72°C for 5 min.

**Table 2:** Newly designed primers for three largest amplicons from Huynh et al. 2008

**Locus**	**Amplicon size**	**F primer (5**^**′**^**-3**^**′**^**)**	**R primer (5**^**′**^**-3**^**′**^**)**
VNTR 2	178 bp	cgctctcctcgcccgcttcttctt	tgtttttggttgcgcatggccg
VNTR 29	150 bp	gtttgtcgtcgcgggagagattagggg	cggcaggcgcttgaggatgagg
VNTR 33	213 bp	cggataggcgcggcgtgagtaagg	acaacatggcgcgtgaaaggccc

VNTR PCR amplicons were discriminated through electrophoretic analysis with an ABI PRISM 3130xl automated fluorescent capillary DNA sequencer (Applied Biosystems Inc., Foster City, CA). All samples were run in triplicate with three different PCR steps each as well as on different fragment analysis runs to examine the reproducibility of the analyses. Each run contained validated *B. melitensis* and *B. suis* DNA standards as positive controls. Fragment analysis was performed with GeneMapper software (Applied Biosystems). Fragment sizing was accomplished by comparison to a 1200 base LIZ-labelled size standard (Applied Biosystems). Customized bins for allele calls in GeneMapper allowed automated scoring of the VNTR alleles. When isolates had more than one amplification peak (i.e. multiple alleles at a locus), we chose the highest peak as the main allele but also recorded alternate alleles. Phylogenetic relationships derived from complete MLVA genotypes were determined using maximum parsimony algorithm in PAUP 4.0 Beta 10 (Sinauer Associates Inc., Sunderland, MA).

## Results

**Figure 1:**
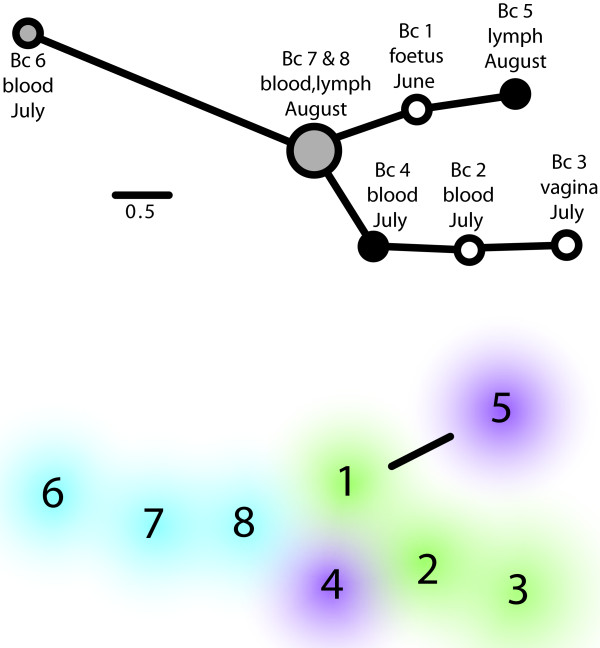
**MLVA-15-based phylogeny. a**) MLVA-15-based phylogram of *Brucella canis* isolates from the affected kennel. Scale bar indicates number of substitutions per position. Circle colour indicates isolates from same individual. **b**) Cloud phylogeny indicating the overlap among primary and alternate alleles. All isolates except Bc 5 share primary and alternate alleles. Cloud colour indicates isolates from same individual.

Ten of the 15 MLVA loci did not mutate in any of the strains (Table 1). VNTR 3 had the same allele for 7 isolates and had a null allele for 1 isolate so was considered monomorphic in our analyses. For polymorphic loci, VNTRs 1 and 30 had single repeat insertions in isolate Bc 6. Both VNTR 2 and 33 were highly variable among the eight isolates and demonstrated multiple repeat changes. Alternate alleles occurred in three of these loci, VNTRs 1, 2, and 33. In 8 of 10 instances the alternate allele was present as the primary allele in at least one other isolate. Phylogenetic analysis of the eight samples placed the isolates onto three branches with genetic differences among the strains within each branch (Figure 1a). Six of the eight isolates had unique genotypes. Strains from the same dog but taken at different dates or collection sources occurred on different branches. For instance, strain Bc 1 appears more closely related to strains Bc 4, 5, 6, 7 and 8 than it does to Bc 2 and 3, although Bc 1, 2, and 3 all came from the same female dog. Isolates Bc 4 and 5 came from the same stud dog a month apart but were more closely related to other isolates on the tree from different dogs. Isolate Bc 6 from the puppy (from another female than the one described here) was genetically quite distinct from any other isolates and had different alleles at VNTRs 1 and 30. Interestingly, Bc 6 was collected a month earlier than two additional isolates from this dog (Bc 7 & 8).

Including alternate alleles in the analysis by using clouds instead of circles and lines allows illustration of the high degree of overlap among primary and alternate alleles (Figure 1b). For VNTR 1, the primary allele for Bc 6 was shared with Bc 2 & 8. VNTR 2 had the primary allele for Bc 1, 4, 7, & 8 shared with Bc 2 & 6. Finally, VNTR 33 had Bc 2 & 4 share an allele with Bc 1 and the primary allele in Bc 3 was shared with Bc 6, 7 & 8. Thus, there was considerable overlap among genotypes when primary and alternate alleles were evaluated.

## Discussion

MLVA provided discrimination among the otherwise indistinguishable isolates from different animals and from within each host, with VNTR mutations being detected at multiple dates and tissue sites. It is strongly suspected that the strains isolated from the dogs originated from the same recent introduction to the kennel, with subsequent rapid *in vivo* mutations. Strain Bc 1 was isolated from the foetus of the female in June. This isolate is distantly related in the tree to Bc 2 & 3 originating from the blood and vagina, respectively, from that female in July. Since these genotypes are closely related to the rest of the isolates and these isolates came from a foetus, the female parent was almost certainly infected as well. Additional *in vivo* evolution route also occurred in the stud dog with VNTR mutations between the collections of the July (Bc 4) and August (Bc 5) isolates. Similar *in vivo* evolution was observed in the case of the isolates (Bc 6-8) of the female puppy as well. These results are consistent with rapid within-host evolution of *Burkholderia pseudomallei* in acute infections in humans [9], demonstrating that VNTR changes can happen within weeks, as well as data from *Yersinia pestis* in fleas their prairie dog hosts that are consistent with within host evolution [20]. High mutation rates are inferred for several of the loci we used in our MLVA. Although Huynh et al. [5] found that average allelic diversity for *B. suis* (of which *B. canis* is a distinct lineage within it) was a moderate 0.49, allelic diversity in VNTRs 2, 3, 1, 33, 30 was quite high at 0.77-0.88, with the number of alleles per locus ranging from 6-13, indicating that these loci account for much of the diversity seen over short time periods. We must note that although minimal generations occurred during culturing during the short incubation period, we cannot rule out some mutations occurring *in vitro*. Direct MLVA testing on samples prior to culturing is recommended in future studies to rule out this possibility, although the number of generations within the dogs would be expected to be much higher than what is typical under standard culturing. We also note that the variation we are detecting is for neutral genetic markers, those presumably not under selection, and thus do not know what changes may be occurring in loci potentially under selection such as those involved in pathogenicity.

The emergence of alternate alleles that become primary alleles shows the fluidity of genetic variation when assessed by MLVA and are a particularly important aspect of this study. Alternate alleles are traditionally ignored as noise in phylogenetic analyses. Rather than making the determination of relationships among isolates more confusing, however, alternate alleles reveal the potential mutations that connect isolates. Nonetheless, a simple depiction of the relatedness of the isolates was not possible in this study. Several of the loci were so variable that determining the direct relationship of isolates was challenging since isolates from the same animal were not always adjacent to each other on the tree. High amounts of mutations at the most variable loci and the subsequent occurrence of homoplasy reduces the utility of VNTRs for some investigations, as seen in *B. melitensis*[21] and leading to lowered weights of these types of loci in analyses [22]. If the most variable markers are excluded from analyses however, then few or no isolates are distinguishable since variation is exclusively within the most polymorphic loci. There is a fine line between adding more polymorphic loci and having that addition variation being less reliable. The application of the study is that rapid within-host evolution is occurring in *Brucella* infections and that we can detect it. However, this evolution is so rapid for some genetic markers that complete knowledge of the relationships of various isolates is not clear, especially for isolates collected from outbreak cases, so that fine-scale epidemiological studies are not possible in all instances.

## Conclusions

The current study is the first to employ fine-scale genotyping on a comprehensive *in vivo* isolate collection derived from a *B. canis* outbreak. This investigation highlights the rapid genetic changes of several VNTRs of *B. canis* and the emergence of alternate alleles at some loci but also shows that MLVA was not an ideal method to analyse the epidemiological relationship of strains in the same outbreak. Nonetheless, the ability for *B. canis* to diversify over a short time frame has substantial implications for our understanding of infection caused by *B. canis*.

## Abbreviations

VNTR: Variable number tandem repeats analysis; MLVA: Multiple locus variable number tandem repeats analysis.

## Competing interests

The authors declare that they have no competing interests.

## Authors’ contributions

MG conceived of the study and with JTF wrote the manuscript and analysed the data. BDR and CAA performed the genotyping. SJ performed the bacteriological examination. PSK helped write the manuscript. All authors have read and approved the final manuscript.
